# Monitoring of antipsychotic plasma levels in the assessment of poor response and nonadherence to antipsychotics in delusional disorder

**DOI:** 10.1192/j.eurpsy.2021.426

**Published:** 2021-08-13

**Authors:** A. González-Rodríguez, A. Guàrdia, A. Alvarez Pedrero, M. Betriu, J. Cobo, N. Sanz, S. Acebillo, J.A. Monreal, D. Palao Vidal, J. Labad

**Affiliations:** 1 Mental Health, Parc Taulí University Hospital. Autonomous University of Barcelona (UAB). I3PT, Sabadell, Spain; 2 Department Of Mental Health, Parc Taulí University Hospital. Autonomous University of Barcelona (UAB). I3PT. CIBERSAM, Sabadell, Spain; 3 Mental Health, Parc Taulí University Hospital. Autonomous University of Barcelona (UAB). I3PT, CIBERSAM, Sabadell, Spain; 4 Mental Health, Hospital of Mataró. Consorci Sanitari del Maresme. CIBERSAM., Mataró, Spain

**Keywords:** Delusional disorder, Antipsychotic plasma levels, psychosis, Antipsychotics

## Abstract

**Introduction:**

Over the last decades, antipsychotic plasma levels have been used to evaluate therapeutic response, adherence and safety of antipsychotics in schizophrenia. Their clinical utility in delusional disorder (DD) has been poorly studied.

**Objectives:**

To investigate the relationship between plasma concentrations of risperidone (R), 9-OH-risperidone (9-OH-R) and olanzapine (OLZ), and clinical outcomes in DD.

**Methods:**

Case-series of inpatients and outpatients with DD receiving treatment with risperidone (n=19) or olanzapine (n=2). Determination of R, 9-OH-R (active metabolite) and OLZ levels were obtained by high-performance liquid chromatography with electrochemical detection. Clinical variables such as treatment response or adverse events were recorded for all patients. These variables were correlated with two plasmatic ratios in patients treated with R: R:9-OH-R concentration ratio and total concentration-to-dose (C: D) ratio, indicating CYP2D6 activity and R elimination respectively.

**Results:**

Twenty-one patients were included: inpatients (n=10) and outpatients (n=11). Dose range: R, 1-6 mg/day; OLZ, 5-10 mg/day. Three outpatients (R, n=2; OLZ, n=1) presented antipsychotic levels under the detection limit (non-adherence). All R patients showed CYP2D6 activity (R: 9-OH-R ratio <1). Eight patients presented C: D > 14, indicating a reduction of R elimination, which was associated with poor clinical response (n=3), adverse events (n=3) and no clinical relevance (n=2). OLZ (n=2), no association between levels and clinical outcomes.

**Conclusions:**

The determination of antipsychotic plasma levels may be of clinical utility in the assessment of treatment resistance, antipsychotic-adverse events or non-adherence in inpatients or outpatients with DD. Therapeutic drug monitoring should be further studied in future works.

**Disclosure:**

AGR has received honoraria, registration for congresses and/or travel costs from Janssen, Lundbeck-Otsuka and Angelini.

